# Major and Trace Elements of Baobab Leaves in Different Habitats and Regions in Sudan: Implication for Human Dietary Needs and Overall Health

**DOI:** 10.3390/foods13121938

**Published:** 2024-06-19

**Authors:** Abdelhakam Esmaeil Mohamed Ahmed, Massimo Mozzon, Ali Omer, Ayaz Mukarram Shaikh, Béla Kovács

**Affiliations:** 1 Faculty of Agriculture, Food Science & Environmental Management, Institute of Food Science, University of Debrecen, Böszörményi út 138, 4032 Debrecen, Hungary; ahmed.abdelhakam@agr.unideb.hu (A.E.M.A.); ayaz.shaikh@agr.unideb.hu (A.M.S.); 2 Doctoral School of Nutrition and Food Sciences, University of Debrecen, Böszörményi út 138, 4032 Debrecen, Hungary; 3 Faculty of Forestry, University of Khartoum, Khartoum North 13314, Sudan; ali.haroon.ali.omer@univie.ac.at; 4 Department of Agricultural, Food and Environmental Sciences, Università Politecnica delle Marche, Via Brecce Bianche 10, 60131 Ancona, Italy; m.mozzon@staff.univpm.it; 5 Division of BioInvasions, Global Change & Macroecology, Department of Botany and Biodiversity Research, University of Vienna, Rennweg 14, 1030 Vienna, Austria; 6 Young Scientist, World Food Forum, I-00100 Rome, Italy

**Keywords:** cardiotoxicity, micronutrients, calcium, iron, Kordofan

## Abstract

The metabolic needs of the human body and preventing infections require a diet with sufficient amounts of essential nutrients. This study aimed to investigate the importance of Baobab (*Adansonia digitata* L.) dried leaves as a healthy food source by determining the content of macro and trace elements in different habitats and regions. This study was conducted in Sudan and covered three different habitats, wetland (W), plainland (P), and mountain (M), in two regions (Blue Nile and Kordofan). The dry matter (DM) of Baobab leaves was considered for analyzed menials, and the results showed that the mean values were significantly affected by habitats where Baobab trees grew. The highest contents of potassium K (1653 ± 34 mg/100 g) and sodium (Na) 7.67 ± 1.18 mg/100 g were found in the W zone, whereas the highest contents of calcium (Ca) 2903 ± 187 mg/100 g and magnesium (Mg) 529 ± 101 mg/100 g were detected in the M and P zones, respectively. In addition, the two regions showed significant differences in trace and macro elements, i.e., higher levels of iron (Fe) 17.17 ± 2.76 mg/100 g and magnesium (556 ± 55 mg/100 g) were found in the Kordofan region while higher levels of zinc (Zn) 2.548 ± 0.55 mg/100 g and calcium (2689 ± 305 mg/100) were in the Blue Nile region. These varying amounts of elements can be used in our daily diets because of their potentially healthy effects, especially in areas where access to nutrient-rich foods is limited.

## 1. Introduction

**Figure 1:**
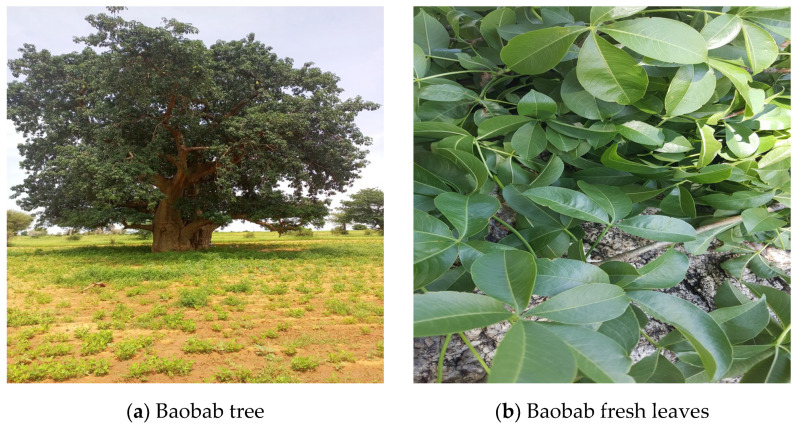
Baobab tree found in plain land, Kordofan, Sudan (**a**); Baobab fresh leaves obtained from Baobab tree found in Mountain, Kordofan, Sudan (**b**).

Eating a healthy diet that contains all essential nutrients in sufficient quantities is considered one of the most effective ways to meet our energy needs and prevent non-communicable diseases [1]. The most important macro elements for human nutrition are (Ca), potassium (K), sodium (Na), and magnesium (Mg), while iron (Fe), copper (Cu), zinc (Zn), and manganese (Mn) are categorized as trace elements [2]. Therefore, a sufficient supply of these elements can help to maintain the health of the human body [3,4]. A public EU Register of Nutrition and Health Claims lists all permitted nutrition claims and all authorized health claims [5] in accordance with the provisions of Regulation (EC) No 1924/2006, thus ensuring full transparency for consumers and food business operators. The study conducted by Amiot et al. has indicated that plant-based foods are essential for providing nutritional patterns [1]. Therefore, the discovery of a new plant resource with a healthy diet is needed in the face of global food shortages [6]. The Baobab plant *Adansonia digitata* L. is a remarkable example of providing an enriched human diet with important elements and nutrients [7]. The Baobab is a massive tree with a diameter of approximately 10 m that can grow up to a height of 25 m (Figure 1a, Baobab tree).

Baobab trees grow in the humid and arid regions of tropical zones [7]. In Sudan, Baobab trees are mainly distributed in the south-eastern region of the country on sandy, loamy, and rocky soils, typically in savannahs with an abundance of short grasslands and the mountain topography in central Sudan [8,9]. Baobab trees are common in the extremely dry regions of Darfur and Kordofan and thrive, especially along wadis, where water accumulates during the rainy season [8]. Baobab is a multipurpose tree that provides a variety of products with different uses for food and medicinal purposes [10,11,12]. It also provides numerous products, such as fruit pulp, seeds, and leaves, which are mainly used as natural foods and consumed daily by the rural population in Africa when food is scarce [12,13]. The Baobab tree can provide approximately 130 kg of fresh leaves annually (Figure 1b, Baobab fresh leaves) and can be dried and consumed as a valuable source of nutrients [14,15]. Therefore, an assessment of the nutritional value of Baobab leaves could reveal the overall benefits of this tree, especially in regions where food is scarce.

The only Baobab product authorized for consumption and trade in the EU market is dried fruit pulp, which is recognized as a novel food under Regulation (EU) 2015/2283 [16]. However, the Baobab tree has several other useful parts with high nutrient contents. Some studies have identified Baobab leaves as a product with a high content of macro- and micronutrients [17]. Baobab trees are found in different African countries and regions of Sudan, but the nutritional content and variability of leaf compositions in these areas are not well known [18].

This study aimed to investigate the potential of Baobab leaves as a healthy food for humans by determining the content of macro elements (i.e., sodium, potassium, calcium, and magnesium) and trace elements (i.e., copper, manganese, iron, and zinc). The specific objective of this study was to assess the variation in the concentrations of these minerals in different habitats and regions in Sudan.

## 2. Materials and Methods

### 2.1. Sample Site and Collection

**Figure 2:**
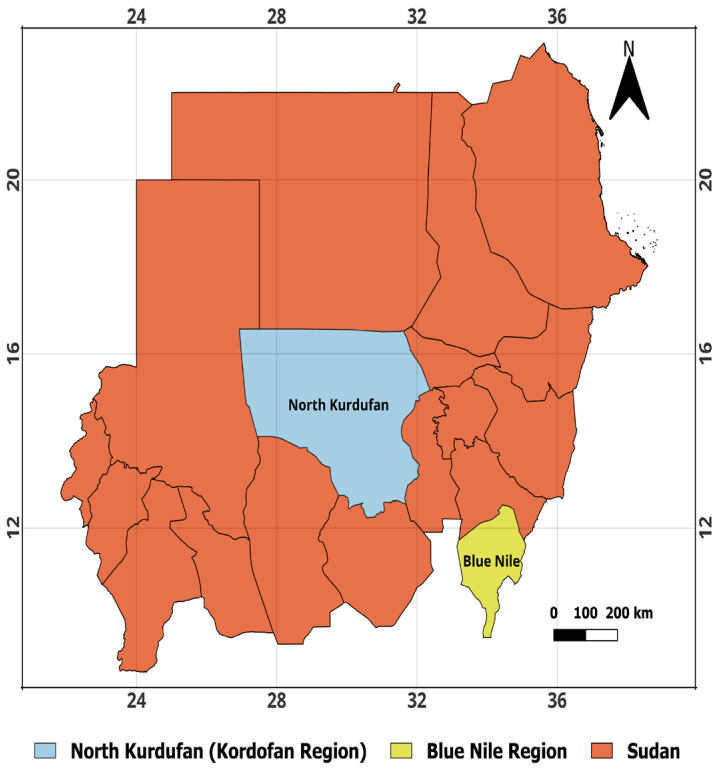
Regions where Baobab leaves were collected in Sudan. Source, QGIS 3.20.1 software.

**Figure 3:**
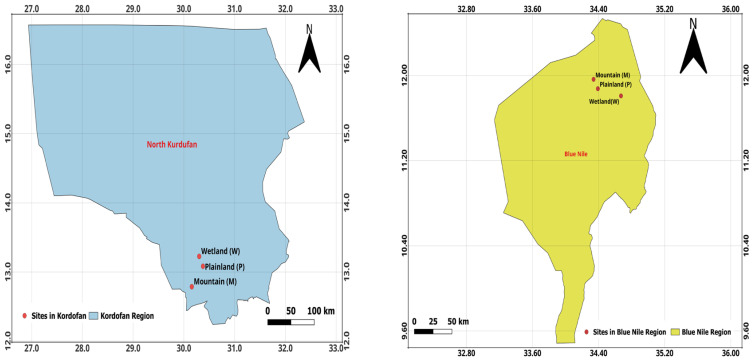
Study sites in Kordofan and Blue Nile regions. Source, QGIS 3.20.1 software.

For this preliminary study, Baobab leaves samples were collected in Sudan between 15 July 2022, and 15 September 2022. Two regions were covered—the Blue Nile in southeastern Sudan (with coordinates Latitude: 11°15′00″ N and Longitude: 34°10′00″ E) and North Kordofan, a dry zone in central Sudan (with coordinates Latitude: 14°68′33″ N and Longitude: 29°93′33″ E)—as shown in Figure 2. Three habitats were chosen for each region—wetland (W), plainland (P), and mountain (M)—as shown in Figure 3. QGIS 3.20.1 software was used to create maps that show areas where Baobab leaf samples were collected. Each area covered approximately 1500 km^2^. Ten trees were selected from each site, and two samples of fresh Baobab leaves were collected per tree, amounting to 3 kg of leaves per tree, to ensure representative sampling. These samples were dried under a shed to approximately 93% dry matter and were completely packaged in polyethylene bags. Approximately 18 samples weighing 500 g each were moved to Debrecen, Hungary, for elemental analysis.

### 2.2. Baobab Leaves Sample Preparation

Baobab leaves were manually cleared of any extraneous matter, followed by air-drying. Subsequently, they were ground using an electric grinder with a mesh size of 10. The resulting powder was stored in labeled sanitary plastic containers until later used.

### 2.3. Mineral Analysis

Mineral analysis was conducted at the Institute of Food Science laboratory at the University of Debrecen, Hungary. The dried matter (DM) of Baobab leaf powder was analyzed using inductively coupled plasma–optical emission spectrometry (ICP-OES) according to the protocols conducted by [19]. The specific instrument used for the analysis was the iCAP 6300 from Thermo Fisher Scientific, based in Waltham, MA, USA.

Mineral analysis involves several steps, with 1 g of dried baobab leaves being subjected to ashing at 550 °C overnight. The resulting ash was then dissolved in 30 mL of 3 M nitric acid and left for 72 h to ensure complete dissolution of the minerals. After this period, the acid solution was thoroughly mixed and filtered through filter paper to remove any solid impurities.

An acid solution containing dissolved minerals was then used for the instrumental measurement of various elements in (mg/100 g) DM, including Ca, Mg, K, Na, Cu, Fe, Mn, and Zn. Appropriate standard solutions were prepared for each metal used in the analysis to ensure accurate calibration and quantification.

### 2.4. Statistical Analysis

Data analysis was conducted using R programming environment (version 3.6.1, R Core Team). Initially, descriptive statistics (mean and standard deviation) were calculated for each trace and macro element across the different habitats and regions. To test the differences in leaf elements between the different habitats, an analysis of variance (ANOVA) was performed. This was followed by the Tukey HSD (Honest Significant Difference) post hoc test to identify specific differences between the groups. A two-sample t-test was conducted to test the differences in leaf elements between the two regions.

Multivariate analysis (two-way (factorial) ANOVA) was performed using JMP^®^ version 10 (SAS Institute Inc., Cary, NC, USA) to test and estimate the effect of the independent factors (regions and habitats) and their interaction on the measured variables (macro- and micro elements). The significance level was set at *p* < 0.05. A reduction in variables was achieved by conducing principal component analysis (PCA) on the correlation matrix to describe the relationships between the analytical data and the geographical origin of the samples (regions and habitats).

## 3. Results

### 3.1. Regional Differences

**Figure 4:**
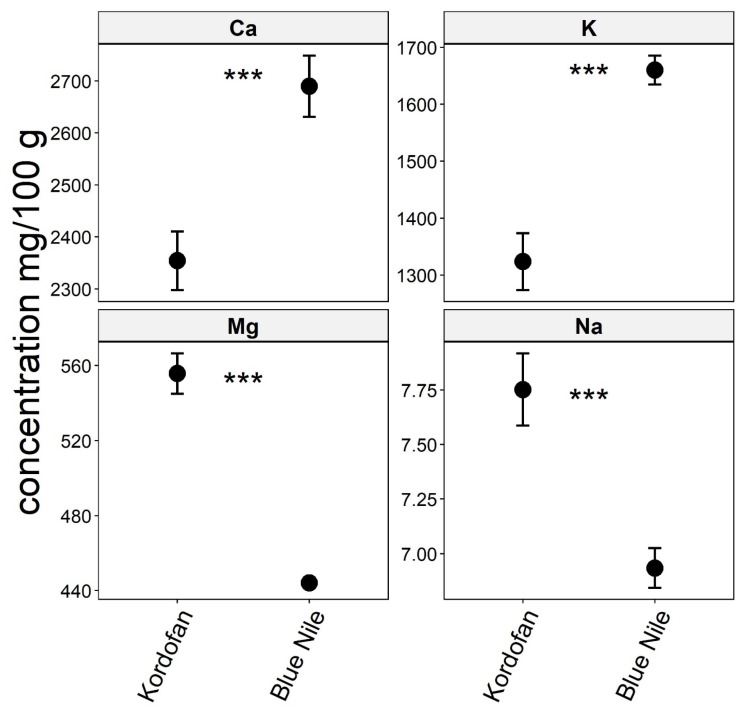
Macro elements (Ca, K, Mg, and Na) concentration of Baobab leaves in two regions, Blue Nile and Kordofan, Sudan. Significance codes: ‘***’ 0.001.

The results revealed that the levels of calcium and potassium in Baobab leaves were higher in the Blue Nile (2689 ± 305 mg/100 g and 1660 ± 132 mg/100 g), whereas, in the Kordofan region, there were greater levels of magnesium and sodium (556 ± 55 mg/100 g and 7.75 ± 0.85 mg/100 g; see Supplementary Materials (Table S1)). Using a two-sample t-test at a 5% level of significance, the true difference in the mean concentrations of macro elements (Ca, K, Mg, and Na) significantly differed between the two regions (*p* < 0.001) (Figure 4).

**Figure 5:**
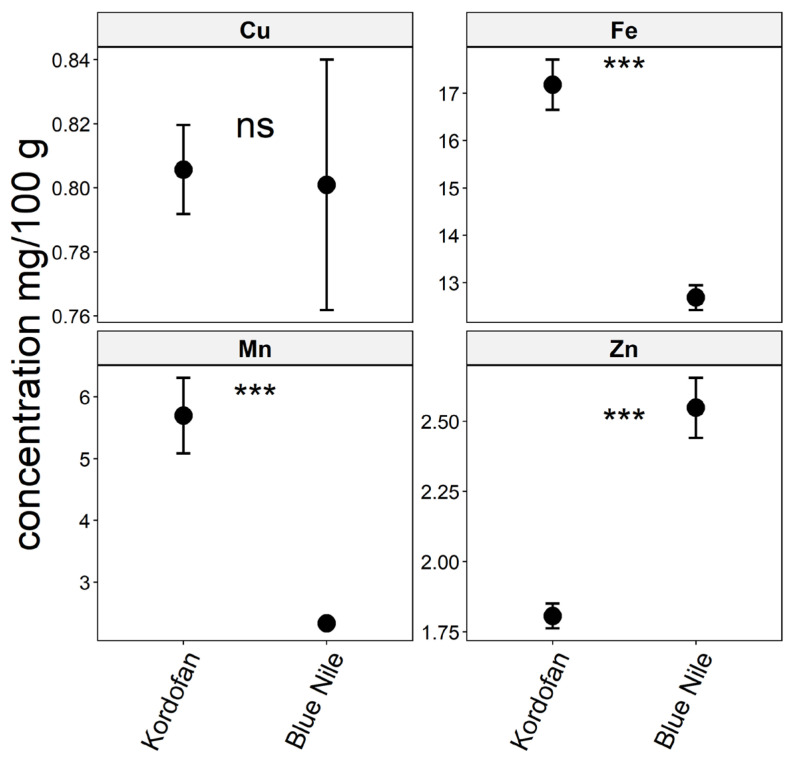
Trace elements (Fe, Cu, Mn, and Zn) concentration of Baobab leaves in two regions (Blue Nile and Kordofan) in Sudan. Significance codes: ‘***’ 0.001 and ns: not significant.

Additionally, the analysis of trace element concentrations in the Baobab leaves indicated that there were higher levels of iron and manganese observed in Kordofan (17.17 ± 2.76 mg/100 g) and 5.69 ± 3.17 mg/100 g) and a higher level of zinc in the Blue Nile region (2.548 ± 0.55 mg/100 g) (Table S2). However, these two regions had the same levels of Cu. The results of the two-sample t-test for Fe, Mn, and Zn indicated that there were significant differences (*p <* 0.001) in the data being analyzed, except for Cu (*p* = 0.9094), suggesting that the true difference in means of copper concentration level was not significantly different between the two regions (Figure 5).

### 3.2. Differences between Various Habitats

**Figure 6:**
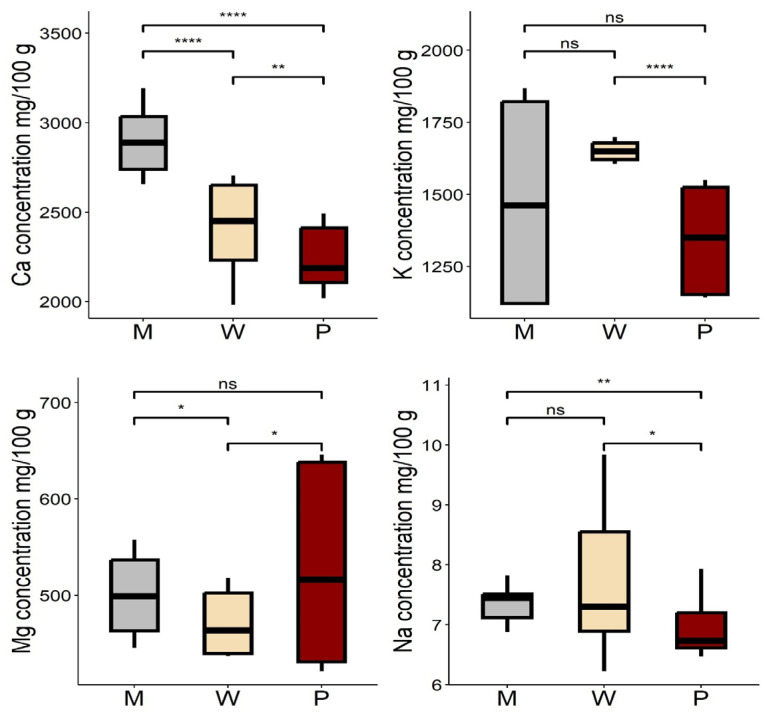
Macro element concentrations of Baobab leaves in different habitats M: mountain, W: wetland, and P: plainland, Sudan. Ca, K, Mg, and Na represent calcium, potassium, magnesium, and sodium. Significance codes: ‘****‘0.0001 ‘**’ 0.01 ‘*’ 0.05, ns: not significant.

In the analysis of macro element concentrations in Baobab leaves among the different habitats, the highest levels of calcium (2903 ± 187 mg/100 g), potassium (1653 ± 34 mg/100 g), magnesium (529 ± 101), and Na (7.67 ± 1.18) were found in the M, W, and P zones, in that order (Table S3). The ANOVA test revealed that the concentrations of all macro elements studied in all three habitats were significantly different (*p <* 0.05) at a 5% significance level. Furthermore, the Tukey HSD multiple comparison test of means showed that there were significant differences (*p <* 0.05) in Ca levels between each pair of study habitats. For Mg and Na, the test also showed a significant difference (*p <* 0.05) between the study habitat pairs (W-P). Similarly, K levels were found to be significantly different between the ecological zone pairs (W-M and W-P) (Figure 6).

**Figure 7:**
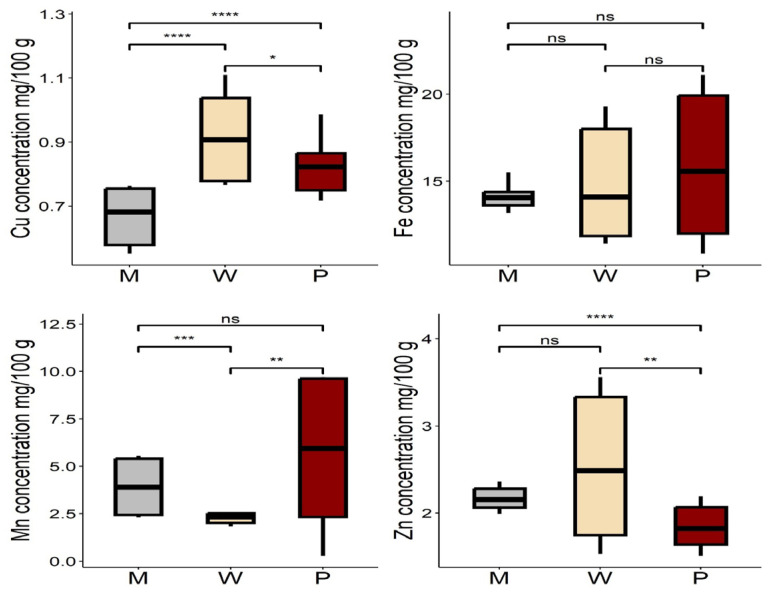
Trace element concentrations of Baobab leaves in different habitats M: mountain, W: wetland, and P: plainland, Sudan. Fe, Cu, Mn, and Zn represented iron, copper, manganese, and zinc. Significance codes: ‘****‘0.0001 ‘***’ 0.001 ‘**’ 0.01 ‘*’ 0.05, ns: not significant.

Among the trace elements in Baobab leaves studied in various habitats, the highest levels of copper (0.91 ± 0.14) and zinc (2.51 ± 0.81) were obtained in ecological zone W, and the greatest amounts of iron and manganese were found in ecological zone P (Table S4). The ANOVA test indicated that the mean concentrations of all trace elements, except iron, were significantly different (*p <* 0.05) within the different habitats (W, P, and M). Tukey’s HSD multiple comparison test of means showed a significant difference (*p <* 0.05) in the levels of Cu among all pairs of habitats, Mn in the pairs (P-M, W-P), and Zn in the W-P pair only (Figure 7).

### 3.3. Multivariate Analysis

Similar to the univariate analysis, the multivariate analysis showed a significant influence of the region and habitat on the mineral content of the Baobab leaves, except for the “Region” factor regarding the Cu content and the interaction of “Region*Zone” for Ca content (Table 1).

**Figure 8:**
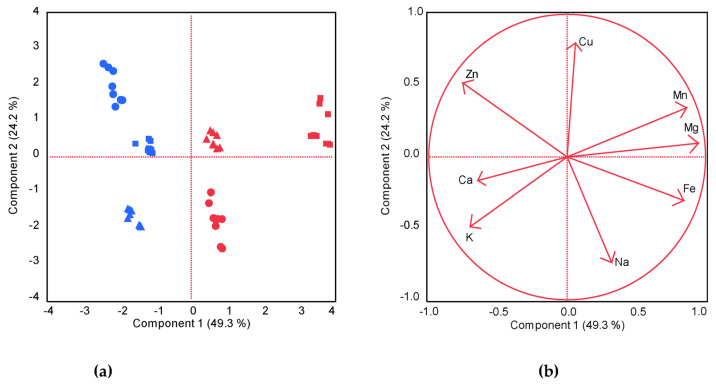
PCA score plots of the Baobab leaves samples (**a**) and PCA loadings plot of the variables on the first two components (**b**). Regions are in blue (Blue Nile) and red (Kordofan). Zones are in circles (W), squares (P), and triangles (M).

Principal component analysis (PCA) was used to evaluate the relationships between the analytical characteristics of the samples and their geographical origins (region, zone). The first two PCs explained 73.5% of the variance in the samples. The loading plot in (Figure 8b) shows that the concentrations of Mn, Mg, and Fe had the highest positive loadings on PC1, whereas Zn, Ca, and K had negative loadings. PC2 was influenced mainly by Cu (positive loading) and Na (negative loading). Positive linear correlations (vectors pointing in the same direction) were found between Mg and Mn (r = 0.179), and between Mg and Fe (r = 0.8101). The strongest inverse correlation (variables arranged in opposite directions with respect to the origin of the axis and far from the origin of the plot) was found between Mn and K (r = −0.8045). The scores plotted in (Figure 8a) show the distribution of samples on the plane defined by PC1 and PC2. PC1 was effective in discriminating between geographic regions: higher levels of Fe (13.65–18.00 vs. 11.79–14.47 mg/100 g), Mn (2.04–5.42 vs. 2.06–2.53 mg/100 g), and Mg (502–626 vs. 431–460 mg/100 g) pulled the samples collected in the Kordofan region to positive loadings on PC1. The samples from the Blue Nile region showed negative loadings on PC1, mainly due to higher levels of Zn (2.09–3.29 vs. 1.60–2.09 mg/100 g), Ca (2380–3071 vs. 2092–2736 mg/100 g), and K (1524–1831 vs. 1122–1683 mg/100 g) than the samples from Kordofan. PC2 was able to differentiate the samples by zone but in different ways for the samples collected from different regions. The P and M samples from Kordofan and the P and W samples from the Blue Nile had positive loadings on PC2, while higher Na contents and lower Cu contents, resulting in negative loadings on PC2 for the W samples from Kordofan and the M samples from the Blue Nile.

## 4. Discussion

Previous research has shown that the Baobab tree plays an important role in increasing the incomes of rural people in Sudan [20], and it is a significant resource for nutrients, with an impact on human health [21]. Baobab leaf extract has effectively reduces doxorubicin cardiotoxicity due to its antioxidative compounds [22]. Previous studies have also shown that the levels of minerals in Baobab leaves within and between different areas in African countries vary significantly [23,24]. Similarly, this study also showed notable variations in several elemental levels, such as potassium, sodium, magnesium, calcium, manganese, and zinc, emphasizing the dynamic nature of the chemical structure of Baobab (Adansonia digitata) leaves. This research adds to the existing knowledge by exploring the effect of habitats and regional factors on the elemental composition of Baobab leaves and discloses a novel perspective on the variability of elemental concentrations, as follows:

### 4.1. Elemental Level Variations

This study highlighted substantial variations in the elemental concentrations of Baobab tree leaves across different regions and habitats. The findings of this study emphasize the importance of considering geographical factors when developing dietary recommendations, particularly for populations living in diverse habitats or areas with lower nutrient levels. By accounting for these variations, dietary advice can be personalized to address the specific nutrient deficiencies prevalent in various geographic locations. Including Baobab leaves in our diets may offer a potential solution for individuals residing in regions with nutrient deficiencies as are a rich source of essential nutrients. More importantly, studies focused on the soil type of Baobab growth as well as regional and ecological differences contribute to the existing literature on Baobab tree products and their value, providing valuable insights for future research and dietary interventions that will be aligned with what was reported by [23,25,26].

### 4.2. Implications of Human Dietary Needs and Overall Health

In general, macro and trace elements are essential components of human nutrition and health, as recognized by the EFSA (Table 2). According to recent studies, calcium significantly contributes to the maintenance of bone health and is important for cancer prevention. Mg plays a crucial role in muscle contraction and glandular secretion [27]. Sodium and potassium also affect blood pressure regulation [28,29,30,31].

In addition, trace elements are important for supporting ongoing regeneration processes, managing oxidative stress in body tissues, and maintaining a robust immune system to fight pathogens [4,5]. Zinc plays a role in the protein structure and controls gene expression [32]. Iron is an essential element found in proteins such as hemoglobin and myoglobin [27]. Copper is an essential trace element in humans and animals, and its deficiency in the diet has serious long-term consequences [33]. Unlike iron and zinc deficiencies that can lead to significant health issues, a manganese deficiency is rare in humans. However, overexposure to Mn can lead to poisoning and diseases such as liver cirrhosis, polycythemia, dystonia, and Parkinson-like symptoms [34,35]. To avoid any health problems, the recommended daily intake must be consumed (Table 3 and Table 4).

Based on our research findings, we compared the average concentration of all macro elements in dried Baobab leaves to the precisely determine their recommended daily intake (Table 3), concluding that Baobab leaves are suitable for a diverse range of individuals and age groups. Furthermore, Ca and Mg components recorded in Baobab leaves ranged from 2354 to 2903 mg/100 g and 444 to 556 mg/100 g, respectively (Tables S1 and S3). The recommended Ca intake ranged from 280 to 960 mg/day, and Mg ranged from 80 to 300 mg/day for children, adult males and females, and pregnant women (Table 3). When referring to its recommended amount, consuming less than 50 g of dried Baobab powder is enough to consume the required Ca and Mg intake, and 200 g is enough for K. However, for Na, the concentration range was 6.93 to 7.75 mg/100 g in dried Baobab leaves (Table S1) compared to the range of the 110 to 2000 mg/day recommended intake (Table 3). In this regard, Baobab leaves have a low capacity to supply adequate daily Na intake requirements. In this scenario, increased daily consumption is recommended to meet the Na requirements.

If we look at Tables S2 and S4, which show the trace element concentrations of dried Baobab leaves, and Table 4, which shows the recommended amount, we can see that the average amount of copper in dried Baobab leaves ranges between 0.66 and 0.91 mg/100 g, while the daily recommended amount is between 0.4 and 1.6 mg/day. So, the findings indicated that consuming 50 g to 200 g of this dried powder will be enough to meet the daily dietary need for copper for all age groups, including infants, children, males, and females. This also holds for iron, manganese, and zinc. In addition, the different amounts of major and trace elements in dried Baobab leaves may work as important and valuable nutrients. This means that Baobab leaves may be useful for meeting our nutritional needs, especially in places where it is hard to obtain nutrient-rich foods.

## 5. Limitations and Future Directions

Although this study provides a constructive understanding of the elemental composition of Baobab leaves, certain limitations should be acknowledged: Firstly, the investigation of other parameters attributed to the nutritional value in addition to trace and macro elements should be investigated. Secondly, this study had a limited sample size, which means that further studies could include more regions and ecosystems in Sudan and other African countries. In addition, prospective studies could assess the protein content, crude fiber, and antioxidant activities of Baobab leaves and investigate variables affecting their nutrient uptake to clarify their influence on leaf structures, for example, the soil characteristics of Baobab-growing areas.

## 6. Conclusions

In conclusion, our study resulted in regional variations of macro and trace element concentrations in dried Baobab leaves obtained from two regions in Sudan. The level of potassium was higher in the Blue Nile region, with the highest concentrations of magnesium and sodium recorded in Kordofan. The concentrations of trace elements showed significant differences between the studied ecosystems. This research emphasizes the likelihood that Baobab leaves are a natural source of crucial minerals with remarkable effects on our health and needs. By elucidating the changes in elemental constituents in different habitats and regions, we added to the body of knowledge supporting the consumption of Baobab leaves as a nutritional resource in different communities and at different ages. Finally, comprehensive research on the nutritional value of Baobab leaves should be conducted to support food safety authorization committees worldwide in developing standards, specifications, regulations, and legislations to allow Baobab leaves to be traded and consumed as novel foods in the EU.

## Figures and Tables

**Table 1:** Analysis of variance applied to a full factorial linear model in which the “Region” and “Zone” are the factors potentially affecting the mineral contents of Baobab leaves.

	ANOVA (Prob > F)		
	Region	Zone	Region*Zone
Ca	<0.0001	<0.0001	0.2869
K	<0.0001	<0.0001	<0.0001
Mg	<0.0001	<0.0001	<0.0001
Na	<0.0001	0.0002	<0.0001
Cu	0.7806	<0.0001	<0.0001
Fe	<0.0001	<0.0001	<0.0001
Mn	<0.0001	<0.0001	<0.0001
Zn	<0.0001	<0.0001	<0.0001

**Table 2:** Description of the main biological functions of minerals.

Elements	Well-Being and Positive Interactions
Calcium	Needed for keeping bones and teeth healthy as well as regular muscle function, precise nerve signal transmission, supporting the operation of digestive enzymes, and cell division.
Potassium	Sustains the health of the neurological operation system, helps to maintain the regular activity of muscles, and reduces blood pressure.
Magnesium	Maintains necessary energy metabolism, enables the nervous system and muscles to operate effectively, promotes regular protein synthesis, aids in preserving electrolyte balance, and improves normal psychological function.
Sodium	Required for muscle function.
Iron	Contributes to the appropriate generation of hemoglobin and red blood cells, facilitates easier transportation of oxygen throughout the body, and supports the health of the immune system.
Manganese	Supports healthy connective tissue development, protects cells from oxidative stress, and maintains regular energy-producing metabolism.
Zinc	Assists in supporting healthy immune system execution, maintaining suitable testosterone levels in the blood, and promoting excellent fertility and reproduction.
Copper	In cases of insufficient micronutrient levels, copper supplementation can reduce fatigue and exhaustion when combined with B vitamins, iron, magnesium, and vitamin C.

Source: [6].

**Table 3:** Adequate intake (mg/day) for (Ca, K, Mg, and Na) approved by the European Food Safety Authority (EFSA) and dietary Reference Intakes for Sodium (mg/day) approved by the National Academies of Sciences, Engineering, and Medicine, Na (2019).

Age Group	Ca	K	Mg	Na	Na (2019)
7–11 months	280		80	200	110
1–3 years	390	1516–2005	Boys and girls: 170	1100	800
4–10 years	680	1668–2750	Boys and girls: 230	1300	1000
11–17 years	960	2093–3712	Boys: 300	1700	1200
18–24 years	860	2463–3991	Male: 350	2000	1500
25 years and above	750	2463–3991	Female: 300	2000	1500
Adult (Male)	-	2463–3991	-	2000	1500
Adult (Female)	-	2463–3991	-	2000	1500
Pregnant	-	2463–3991	-	2000	1500

Source: [36,37], The ‘-’ symbol in certain cells indicates that there are no data available for that specific category.

**Table 4:** Adequate intake (mg/day) for trace elements determined and approved by the European Food Safety Authority (EFSA).

Age Group	Gender	Copper	Iron	Manganese	Zinc
18 and older	Male	1.6	-	-	9.4 to 16.3
18 and older	Female	1.3	-	-	7.5 to 12.7
7–11 months	Infants	0.4	8	0.02–0.5	2.4
1 to 3 years	Children	0.7	5	0.5	2.4–11.8
3 to <10 years	Children	1.0	5	0.8	2.4–11.8
10 to <18 years	Boys	1.3	8	1.3	7.5 to 12.7
10 to <18 years	Girls	1.1	7	1.9	7.5 to 12.7

Source: [36], The ‘-’ symbol in certain cells indicates that there are no data available for that specific category.

## Data Availability

The original contributions presented in the study are included in the article/Supplementary Material, further inquiries can be directed to the corresponding author.

## Supplementary Materials

The following supporting information can be downloaded at: https://www.mdpi.com/article/10.3390/foods13121938/s1. Table S1: Macro elements concentration (mg/100 g) (mean ± SD; *n* = 9) of Baobab leaves samples collected from two regions, Sudan; Table S2: Trace elements concentration (mg/100 g) (mean ± SD; *n* = 9) of Baobab leaves samples collected from two regions, Sudan; Table S3: Macro elements concentration (mg/100 g) (mean ± SD; *n* = 9) of Baobab leaves samples collected from Habitats, Sudan; Table S4: Trace elements concentration (mg/100 g) (mean ± SD; *n* = 9) of Baobab leaves samples collected from Habitats, Sudan.
