# Supplementary material for: Major and Trace Elements of Baobab Leaves in Different Habitats and Regions in Sudan: Implication for Human Dietary Needs and Overall Health
Source: Foods. 2024 Jun 19;13(12):1938. doi: 10.3390/foods13121938 (PMC11202836; doi:10.3390/foods13121938)
Supplement: Supplementary file 1 [file foods-13-01938-s001.zip › foods-3045095-supplementary.pdf]

Supporting material

Table S1. *Macro elements concentration (mg/100 g) (mean ± SD; n = 9) of Baobab leaves samples collected from two regions, Sudan*

Elements	Blue Nile	Kordofan
**Ca**	2689±305	2354±293
**K**	1660±132	1323±259
**Mg**	444±14	556±55
**Na**	6.93±0.47	7.75±0.85

Table S2. *Trace elements concentration (mg/100 g) (mean ± SD; n = 9) of Baobab leaves samples collected from two regions, Sudan*

Trace Elements	Blue Nile	Kordofan
**Cu**	0.80±0.20	0.80±0.07
**Fe**	12.68±1.37	17.17±2.76
**Mn**	2.33±0.42	5.69±3.17
**Zn**	2.548±0.55	1.80±0.23

Table S3. *Macro elements concentration (mg/100 g) (mean ± SD; n = 9) of Baobab leaves samples collected from Habitats, Sudan*

Elements	Mountain	Plain land	Wetland
**Ca**	2903±187	2236±169	2426±222
**K**	1476±365	1345±185	1653±34
**Mg**	500±41	529±101	471±32
**Na**	7.37±0.31	6.97±0.49	7.67±1.18

Table S4. *Trace elements concentration (mg/100 g) (mean ± SD; n = 9) of Baobab leaves samples collected from Habitats, Sudan*

Elements	Mountain	Plain land	Wetland
**Cu**	0.66±0.09	0.82±0.08	0.91±0.14
**Fe**	14.05±0.62	15.83±4.22	14.89±3.27
**Mn**	3.9±1.55	5.84±3.92	2.28±0.26
**Zn**	2.17±0.12	1.84±0.26	2.51±0.81
